# Whip Rule Breaches in a Major Australian Racing Jurisdiction: Welfare and Regulatory Implications

**DOI:** 10.3390/ani7010004

**Published:** 2017-01-16

**Authors:** Jennifer Hood, Carolyn McDonald, Bethany Wilson, Phil McManus, Paul McGreevy

**Affiliations:** 1 Faculty of Veterinary Science, University of Sydney, Room 206, R.M.C. Gunn Building, Sydney 2006, New South Wales, Australia; carolyn131283@gmail.com (C.M.); bethany.wilson@sydney.edu.au (B.W.); paul.mcgreevy@sydney.edu.au (P.M.); 2 School of Geosciences, University of Sydney, Room 435, F09, Madsen Building, Sydney 2006, New South Wales, Australia; phil.mcmanus@sydney.edu.au

**Keywords:** animal welfare, horseracing, racing integrity, whip rule breaches, whip use

## Abstract

**Simple Summary:**

An evidence-based analysis of whip rule breaches in horse racing is needed to address community expectations that racehorses are treated humanely. The study provides the first peer-reviewed characterisation of whip rule breaches and their regulatory outcomes in horseracing, and considers the relationship between rules affecting racing integrity and the welfare of racehorses in a major Australian racing jurisdiction.

**Abstract:**

Whip use in horseracing is increasingly being questioned on ethical, animal welfare, social sustainability, and legal grounds. Despite this, there is weak evidence for whip use and its regulation by Stewards in Australia. To help address this, we characterised whip rule breaches recorded by Stewards using Stewards Reports and Race Diaries from 2013 and 2016 in New South Wales (NSW) and the Australian Capital Territory (ACT). There were more recorded breaches at Metropolitan (M) than Country (C) or Provincial (P) locations, and by riders of horses that finished first, second, or third than by riders of horses that finished in other positions. The most commonly recorded breaches were forehand whip use on more than five occasions before the 100-metre (m) mark (44%), and whip use that raises the jockey’s arm above shoulder height (24%). It is recommended that racing compliance data be analysed annually to inform the evidence-base for policy, education, and regulatory change, and ensure the welfare of racehorses and racing integrity.

## 1. Introduction

The use of whips in horse racing is increasingly being challenged on ethical, welfare [1,2,3,4,5,6,7,8,9,10,11], social sustainability [10], and legal grounds [8]. Industry proponents argue “acceptable use of the whip … means that the whip is used for safety (of both jockey and horse) or to encourage the horse to perform to its best when in contention” [12] (p. 5). The ability of the whip to achieve these goals remains unproven [8], while there is evidence that striking a horse with a padded racing whip would be at least aversive and at worst, possibly painful [4,13,14]. An Australian study by McGreevy et al. [4] found 83% of whip strikes caused indentations of the skin of the horses whipped, and comparative studies in mice and humans showed such deformation is likely to be detected by cutaneous nociceptors [13], as did a recent study in horses [14].

Increasingly, the community is concerned with the humane treatment of animals and, indeed, there are growing calls for the whip to be banned [15,16]. There has been strong criticism of whip use by some experienced industry observers [17], and RSPCA Australia “is opposed to the use of whips on racehorses for the purpose of enhancing performance as they inflict pain and distress” [18].

Self-regulation of the Australian horseracing industry means Stewards enforce the whip rules, including interpreting and determining penalties. Since 2009, there have been a number of controversial reforms made to whip rules in Australia. While these have sought to restrict the number and type of whip strikes allowed, like the whip rules, they are not evidence-based. Indeed, there is a paucity of published data on whip use in Australia (and worldwide), including regulatory outcomes.

In “Responsible Regulation: A Review of the use of the whip in Horseracing” (UK Review) [19], a statistical analysis of whip offences was provided. In their critique of this report, Jones et al. [8] argued the UK Review was not “a “scientific”, peer-reviewed paper” and concluded “further independent scientific review is needed to reach definitive conclusions about whip use on racehorse welfare”. In its defence, the UK Review at least provided some useful statistics on whip use in the UK that, up until the current study, have not been readily available for racing in Australia.

Our study examined data from Racing NSW Stewards Reports (Stewards Reports) [20] and Racing NSW Race Diary (Race Diary) [21] for 2013 (and for 4 months in 2016) in New South Wales (NSW) and the Australian Capital Territory (ACT), which is a major horseracing jurisdiction in Australia. To the authors’ knowledge, this is the first peer-reviewed study to characterise whip use from a regulatory and welfare perspective.

## 2. Materials and Methods

### 2.1. Data Sources

Stewards Reports [20] 1 January 2013–31 December 2013, and 1 January 2016–30 April 2016, were examined to identify numbers and types of breaches of the Australian Rules of Racing (AR) pertaining to whip use and their regulatory outcomes, in each race at every Racing NSW race meeting. Other information, including Racing NSW Breeder Owner Bonus Scheme (BOBS) [22], prize money details, and lengths of races, was obtained from the Race Diary [21].

### 2.2. Other Terms Used

*BOBS* refers to the BOBS in NSW and includes BOBS Extra incentive scheme [22];

*breach* refers to a breach of the AR whip rules recorded in the Stewards Reports, as distinct from a breach that may have occurred but was not recorded;

*breach code* refers to the code we used to denote each whip rule (see Table 1);

*first breach* refers to the first breach recorded by the Stewards in a start (see below);

*location* refers to whether the race track was at a Country (C), Metropolitan (M), or Provincial (P) location as defined in the NSW Local Rules of Racing (LR); C includes races in the ACT (these were excluded from the BOBS analysis as the BOBS status of races in the ACT is not routinely provided in the Race Diary);

*prize money won* refers to the total prize money won by an individual horse in a race;

*riders* refers to both jockeys and apprentices;

*repeat offender* refers to a rider who has more than one whip rule breach recorded in 2013, and includes those with second breaches (see below);

*Rules of Racing* refers to Racing NSW’s Rules of Racing and includes the AR and the LR [23];

*second breach* refers to a breach following a first breach in a start;

*start* refers to a start by an individual horse in a race;

*the 100-metre (m) mark* refers to the start of the final 100 m before the finishing post;

*total prize money on offer* refers to the total prize money offered for a race (including all place getters);

*whip rules* refers to AR 137A. (1)–(9) current in 2013 (see Table 1 for breach codes used);

*$* refers to Australian dollars.

### 2.3. Data Analysis

Data were analysed using R Core Team statistical and computing software (R Foundation for Statistical Computing, Vienna, Austria) [24]. Associations between categorical variables were explored using χ^2^ tests for independence with a *p* value of less than 0.05 as the cut-off for rejecting the null hypothesis. For some analyses, following the rejection of the null hypothesis, additional pairwise χ^2^ tests were employed using a more stringent *p* value (0.05/m, where m is the number of post-hoc hypotheses being tested) to ensure that the familywise type 1 error rate remained acceptable. χ^2^ tests were undertaken using the “chisq.test” function of *r*, which includes a Yates Continuity Correction for 2 × 2 tables but not for other *i* × *j* tables. When one or more cell in the table of expected frequencies was <5, a Fisher’s exact test was performed instead, using the function “fisher.test”. Where pertinent, adjusted standardised residuals were calculated manually. 

In addition to categorical variables, three continuous variables (race length, prize money won, and total prize money on offer) were examined for an association with breach code of the first breach among starts with breaches. Race length was examined by ANOVA using the r functions “anova” and “lm”. After detecting an interaction between the breach code and whether the start was C, M, or P, the dataset was subset into C, M and P datasets and separate ANOVAs were performed, using a *p* value cut-off of *p* < 0.017. The null hypothesis was rejected for M tracks, leading to post-hoc Tukey testing on this dataset using *r*’s “TukeyHSD” function with a 98.3333% family-wise confidence level. The distribution of prize money won and total prize money on offer were positively skewed with outliers, so a non-parametric Kruskal-Wallis rank sum test was performed using the “kruskal.test” function in r. Because the C, M, and P datasets were analysed separately, a *p* value cut-off of *p* < 0.017 was used.

Descriptive data comparing fines and suspensions imposed in the first four months of 2016 with those in the similar 2013 period were analysed manually.

## 3. Results

### 3.1. Overall Frequency of Breaches in 2013

Between 1 January and 31 December 2013, there were 56,456 starts in 5604 races at 785 race meetings on 122 different tracks at Country (C), Metropolitan (M) and Provincial (P) locations in NSW and the ACT. A breach of the whip rules was reported in 348 starts (0.62%) and, of these, 37 included a second breach (corresponding to 10.63% of starts with breaches and 0.07% of all starts). This equates to a breach or breaches being reported in 332 of the 5604 races (5.92%).

Of the 348 starts with breaches, 317 (91.09%) represented the only horses in their races with a breach or breaches; 28 (8.05%) occurred in races in which one other horse had a breach reported (14 races); and three (0.86%) occurred in one race in which two other horses had a breach reported. Further details are provided in Supplementary Materials Table S1.

### 3.2. Locations of Breaches in 2013

The 348 starts resulting in a breach or breaches occurred at 241 of 785 race meetings (30.70%) and at 64 of the 122 racetracks (52.46%). The 37 starts with second breaches occurred at 37 race meetings at 20 different tracks. Despite hosting 67.34% of starts, C tracks recorded only 57.47% of starts with a breach or breaches. Conversely, M tracks hosted 14.53% of starts and yet recorded 22.13% of starts with a breach or breaches. Further details are provided in Supplementary Materials Table S1.

Using a chi-square test, we revealed an association between the frequency of breaches and whether a track was at a C, M or P location (χ^2^ = 19.9703, *p* < 0.01). Post hoc chi-square testing showed starts at C locations were significantly less likely to result in a breach than starts at P and M locations (χ^2^ = 15.0727, *p* < 0.01) and starts at M locations were significantly more likely to result in a breach than starts at C and P locations (χ^2^ = 15.6525, *p* < 0.01).

### 3.3. Whip Rules Breached in 2013

#### 3.3.1. First Breaches

Table 1 shows the whip rule most commonly breached as a first breach was AR 137A. (5)(a)(ii) (Code 17), which accounted for 45.11% of first breaches. Breaches of AR 137A. (4)(b) (Code 8) accounted for 25.00% of first breaches, while breaches of AR 137A. (4)(c) (Code 9) and AR 137A. (5)(a)(i) (Code 16) accounted for 12.64% and 9.48% of first breaches, respectively. This means 54.59% of first breaches were attributable to whip use prior to the 100 m mark (Codes 16 and 17). No breaches were recorded for several whip rules (see Codes 1–5, 10–12, 15, 18–23).

(i) Location of First Breaches

Table 2 shows breach codes for the first breach of a start classified by C, M, P location of the track at which the start occurred.

(a) Location of Code 8 First Breaches (Whip Use That Raises Arm above Jockey’s Shoulder Height)

The probability of a Code 8 first breach was associated with whether a start occurred at an M, C or P location (χ^2^ = 39.0306, *p* ≤ 0.01). Post-hoc testing revealed starts at M locations were significantly more likely to result in the recording of a Code 8 first breach than starts at a C track location (χ^2^ = 38.5024, *p* ≤ 0.01).

(b) Location of Code 9 First Breaches (Whip Use When Horse Is Out of Contention)

This less common rule breach accounted for approximately 1 in 8 first breaches overall. There was no association between Code 9 breaches and C, M, P locations (χ^2^ = 5.007, *p* = 0.0818).

(c) Location of Code 16 First Breaches (Forehand Whip Use in Consecutive Strides prior to 100 m Mark)

The probability of a Code 16 breach was associated with whether a start occurred at a C, M, or P location (χ^2^ = 9.35, *p* ≤ 0.01). Post-hoc testing revealed starts at C locations were significantly less likely to result in a Code 16 breach than starts at other locations (χ^2^ = 7.6338, *p* ≤ 0.01).

(d) Location of Code 17 First Breaches (Forehand Whip Use on More Than Five Occasions prior to 100 m Mark)

The probability of a Code 17 breach was associated with whether a start occurred at a C, M, or P location (χ^2^ = 17.3068, *p* ≤ 0.01). Post hoc testing revealed starts at C locations were significantly more likely to result in a Code 17 first breach than starts at an M location (χ^2^ = 15.8759, *p* ≤ 0.01).

(ii) First Breaches by Rider Gender

Of 348 starts where a code was recorded as breached, 296 (85.06%) had male riders and 52 (14.94%) had female riders. A Fisher’s exact test, examining whether the relative proportions of breach codes (16, 17, 9, 8, or other) were independent of rider gender, was performed using r. The overall *p* value was <0.0001. This suggests recorded breaches depend on whether the rider was male or female. The observed frequencies of breaches of Code 16 (Forehand whip use in consecutive strides prior to 100 m mark) and Code 8 (Whip use that raises arm above jockey’s shoulder height) are similar to expected under independence. Comparing adjusted standardised residuals to the Z distribution, female riders appeared slightly, but not significantly, overrepresented for codes with breaches other than Code 8, Code 9 (Whip use when horse is out of contention), Code 16, and Code 17 (Forehand whip use on more than 5 occasions prior to 100 m mark) (*p* = 0.095); significantly underrepresented for Code 17 (*p* < 0.001); and significantly overrepresented for Code 9 (*p* < 0.001).

#### 3.3.2. Second Breaches

Of 348 starts with first breaches, 37 (10.63%) had second breaches. The majority (21% or 56.76%) were breaches of Code 16 (Forehand whip use in consecutive strides prior to 100 m mark), while another 11 (29.73%) were breaches of Code 17 (Forehand whip use on more than five occasions prior to 100 m mark). The five remaining second breaches (13.51%) were breaches of Code 8 (Whip use that raises arm above jockey’s shoulder height). This means 86.49% of second breaches occurred prior to the 100 m mark.

(i) Location of Second Breaches

While second breaches occurred in fewer than 10% of first breaches at C and M locations, at P locations this number was significantly higher at 22.54% (χ^2^ = 11.7733, *p* < 0.01). Using a Fisher’s exact test, we found a significant association (*p* = 0.02294) between the C, M, P location of the track and the breach code of a second breach. This association most likely relates to the apparent underrepresentation of breaches of Code 17 (Forehand whip use on more than five occasions prior to 100 m mark) and overrepresentation of breaches of Code 16 (Forehand whip use in consecutive strides prior to 100 m mark) at C locations, especially when compared to P locations. Further details are provided in Supplementary Materials Table S2.

(ii) Second Breaches by Rider Gender

Of 37 starts with second breaches, 34 (91.89%) were by male and three (8.11%) by female riders. Once a first breach was recorded, we found no association between rider gender and a second breach being recorded (χ^2^ = 0.9793; *p* = 0.3224). This suggests that once a first breach is recorded, the frequency of a second breach is independent of gender.

#### 3.3.3. Whip Rules Breached in First and Second Breaches

Code 17 (Forehand whip use on more than five occasions prior to 100 m mark) was the most common whip rule breached in first breaches and Code 16 (Forehand whip use in consecutive strides prior to 100 m mark) the most common second breach. Of 37 starts with two breaches recorded, 19 (51.35%) followed this pattern. The next most common pattern, seen in 10 horses (27.03%), was the reverse of this. This means where two breaches were recorded in one start, over 75% (29 out of 37) were breaches of these two whip rules and as such occurred prior to the 100 m mark. When first and second breaches are considered together, breaches of Code 17 were the most common (44%), followed by breaches of Code 8 (Whip use that raises arm above jockey’s shoulder height) (24%).

### 3.4. Breach Outcomes in 2013

Our examination of the Stewards Reports showed the format varied between racetracks. While all contained the major findings, these were presented differently and in varying detail. For example, some Stewards Reports listed Conviction Recorded next to some whip rule breaches, even though a recorded breach of any whip rule constitutes a Conviction Recorded [25].

The most common outcome for first and second breaches of a whip rule was a reprimand (48.28% and 64.86%, respectively). Where a fine was the outcome, as in 104 cases (29.88%) of first breaches, the most common value (mode) and the median value was $200. There was only one fine ($200) awarded for a second breach (see Table 3).

#### 3.4.1. First Breach Outcomes

All Code 6 first breaches (Excessive/unnecessary/improper whip use) resulted in cautions (*n* = 6) or reprimands (*n* = 4). Breaches of Code 17 (Forehand whip use on more than five occasions prior to 100 m mark), Code 8 (Whip use that raises arm above jockey’s shoulder height), Code 9 (Whip use when horse is out of contention), and Code 16 (Forehand whip use in consecutive strides prior to 100 m mark), were the most common first breaches (*n* = 321), and had variable outcomes. There were only nine suspensions in total and these resulted from breaches of Codes 16 and 17. The median suspension imposed was eight days (range 7–10). Further details are provided in Table 4.

For first breaches, 104 fines were imposed resulting in a total of $25,600. Further details are provided in Supplementary Materials Table S3. When second breaches are included (see Section 3.4.2 below) the total is $25,800. 

#### 3.4.2. Second Breach Outcomes

Second breaches (*n* = 37) were recorded for breaches of Code 16 (Forehand whip use in consecutive strides prior to 100 m mark) (*n* = 21), Code 17 (Forehand whip use on more than five occasions prior to 100 m mark) (*n* = 11), and Code 8 (Whip use that raises arm above jockey’s shoulder height) (*n* = 5). The four suspensions imposed were for Code 16 and 17 breaches. The median suspension imposed was 7.5 days (range 5–14). The only fine imposed for a second breach was $200 (1× Code 17). No cautions were issued for Code 16 or 17 second breaches (this is similar to first breaches in which there was only one caution issued for these breaches). Further details are provided in Supplementary Materials Table S4.

### 3.5. Prize Money in 2013

#### 3.5.1. Prize Money Won by Horses with Breaches

Using the Kruskal-Wallis test, we found horses whose riders had Code 9 breaches (Whip use when horse is out of contention) won significantly less money than horses whose riders breached other codes in races at C, M, and P locations (*p* < 0.0001; *p* = 0.0086; *p* = 0.0002, respectively).

The total prize money won by horses in starts where a first breach resulted in a fine was $1,108,425, and for the single start where a second breach had a fine imposed ($200), the total prize money won was $17,500, making the total prize money won by horses in starts resulting in fines $1,125,925. The median prize money won where there was one breach reported was $1000 (range $0–$105,000); for two breaches it was $2100 (range $0–$400,000), and for all starts with breaches it was $1325 (range $0–$400,000).

Overall, fines represented about 2.29% of the total prize money won by these horses. The median percentage of a fine compared to the prize money won in an individual case was 13.9%. The number ranges very widely from 0.19% (for a horse that won $105,000 and the rider received a $200 fine) to eight cases in which the fine exceeded the prize money won.

#### 3.5.2. Total Prize Money on Offer

(i) Total Prize Money on Offer in Races with Breaches

Breaches occurred in 332 races in which the total prize money on offer was $12,636,450 ($2,860,200 was from 194 C races (*mean = $14,743.30 per race*); $2,014,500 was from 65 P races (*mean = $30,992.31 per race*), and $7,761,750 was from 73 M races (*mean = $106,325.34 per race*)). Overall, fines represented about 0.20% of the total prize money on offer in races with breaches.

The median total prize money on offer in races in which there was one whip rule breach was $15,000 (range $2500–$250,000), for two breaches it was $22,000 (range $6000–$2,250,000), and for all starts with breaches it was $15,000 (range $2500–$2,250,000).

(ii) Total Prize Money on Offer in Races with Breaches by Location

There was no significant (Kruskal-Wallis χ^2^ = 10.3853) association between total prize money on offer for races and the frequency of code breaches at M (*p* = 0.0344), C (*p* = 0.5327) or P locations (*p* = 0.1771). Further details are provided in Supplementary Materials Table S5.

### 3.6. Race Length and Breaches in 2013

Breaches occurred in races ranging from 900 to 2400 m (median 1400 m). Using ANOVA with post-hoc Tukey tests, we found the length of races where there were breaches of Code 8 (Whip use that raises arm above jockey’s shoulder height) or Code 9 (Whip use when horse is out of contention) was significantly lower (*p* < 0.01; *p* = 0.01, respectively) than the length of races where there were Code 17 breaches (Forehand whip use on more than five occasions prior to 100 m mark) at M tracks (but not at C or P tracks). Further details are provided in Supplementary Materials Table S6.

#### Length of Races with Breaches by Type of Race and Whip Rule Breached

Table 5 treats first (*n* = 348) and second breaches (*n* = 37) equally (Total 385), and shows 56.88% of all breaches occurred in sprint races. It also shows that overall Code 17 breaches were the most common.

### 3.7. Riders with Breaches in 2013

#### 3.7.1. Frequency of Riders with Breaches

One hundred and thirty nine riders were responsible for the 348 starts with breaches, and of these, 51.08% were repeat offenders (riders who had more than one whip rule breach recorded in 2013, including those with second breaches in a start). Further details are provided in Supplementary Materials Table S7.

(i) Riders with the Highest Number of Breaches

Of the 15 riders with the highest numbers of breaches, only one was an apprentice and one was female. Further details are provided in Supplementary Materials Table S8.

(ii) Riders Breaching Whip Rules in Two Races at the Same Race Meeting

There were 13 race meetings (3 M, 4 P, 6 C) where the same riders had breaches in two races, with one rider having breaches in two races at two different race meetings. There were no instances of a rider breaching in more than two races at the same race meeting. Four of the riders were apprentices (two males, two females). Seven of these 13 riders were jockeys with the highest numbers of breaches overall and were male. Further details are provided in Supplementary Materials Table S9.

#### 3.7.2. Fines and Prize Money of Riders with Breaches

The $ value of fines imposed on riders for whip rule breaches as a percentage of the prize money won by the horse and as a percentage of the rider’s 4.95% share of the prize money, varied widely (see Table 6).

#### 3.7.3. Whip Rule Breaches by Apprentices and Jockeys

We were unable to consider the overall percentage of riders in 2013 that were apprentices as opposed to jockeys. However, we did investigate whether, among riders with breaches, apprentices were associated with different breach codes than jockeys. A χ^2^ analysis of rider status and breaches of Code 16 (Forehand whip use in consecutive strides prior to 100 m mark); Code 17 (Forehand whip use on more than five occasions prior to 100 m mark); Code 8 (Whip use that raises arm above jockey’s shoulder height); and Code 9 (Whip use when horse is out of contention), plus a pool of the remaining breach codes, suggested that, among riders with breaches, the whip rule breached depends on jockey status (χ^2^ = 29.4424, *p* < 0.0001). Comparing adjusted standardised residuals to the Z distribution, apprentices appear slightly, but not significantly, underrepresented among breaches of Code 8 (*p* = 0.09653) and Code 17 (*p* = 0.0452, which is insufficient after correction for family error rate), and significantly overrepresented among breaches of Code 9 (<0.0001). As there were different breach code patterns depending on C, M, or P location, and because there might be differences in the percentage of jockeys versus apprentices at these locations, we treated this as a potential confounding factor and conducted separate post-hoc Fisher’s exact tests for independence of breach code and rider status on breaches at C (*p* = 0.0003), P (*p* = 0.0008), and M (*p* = 0.4705) locations separately. The apparent overrepresentation of apprentices among Code 9 breaches remained evident at C and P locations in the post-hoc testing.

### 3.8. Race Finishing Positions and Percentage of Whip Rule Breaches in 2013

**Figure 1:**
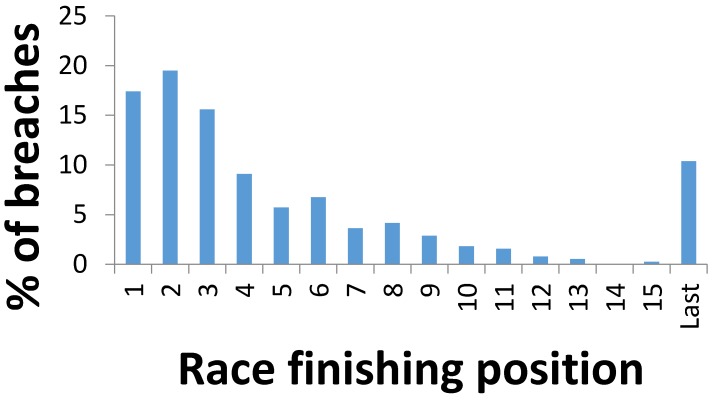
Race finishing positions and percentage of whip rule breaches.

Figure 1 shows the highest percentage of whip rule breaches occurred in horses that ran second, closely followed by horses that ran first. Horses in first place had riders with the highest number of first breaches, but when second breaches were included, second place finishing horses had the highest number of breaches, although the difference between first and second horses was not significant in this sample. The next highest percentage was in horses that ran third. Overall, horses finishing first, second, or third had significantly more breaches than horses finishing in other positions (Chi-squared = 69.4457, *p*-value < 0.0001, if we assume a single first, second, and third finisher in each race of the sample). The next highest percentage was seen in horses that ran last.

When we examined breach types in horses that ran last, we found 26 (65.00%) of the total 40 (39 first breaches and one second breach) were Code 9 breaches.

### 3.9. Breeder Owner Bonus Scheme (BOBS) in 2013

As explained in Materials and Methods, these analyses exclude ACT data.

#### 3.9.1. Frequency of BOBS and Non-BOBS Races by Location

Non-BOBS races were more likely to be held at M locations (over 7 out of 10 Non-BOBS races occurred at M tracks, despite about 15.5% of races being held at these tracks). Further details are provided in Supplementary Materials Table S10. A chi-square test for independence of BOBS/Non-BOBS races and location gives a chi-square statistic of 527.1027 and a *p* value well <0.01.

#### 3.9.2. Frequency of Whip Rule Breaches in BOBS Races

At C locations, 187 (97.91%) of the 191 starts resulting in a breach or breaches were in BOBS races, which comprised over 99% of races. At M tracks, 62 (80.52%) of 77 starts resulting in a breach or breaches occurred in BOBS races, which comprised 82% of races. At P tracks, 67 (94.37%) of 71 starts resulting in a breach or breaches occurred in BOBS races, which comprised 97.39% of races. These data do not suggest BOBS races are more likely to result in breaches. Further details are provided in Supplementary Materials (see Tables S10 and S11, and Section S3.9.2.).

#### 3.9.3. Percentage of Breaches in BOBS Horses

Of 54,380 starts in NSW, approximately two of every nine horses with a breach or breaches were BOBS horses. Our data do not suggest breaches are more likely in BOBS horses in BOBS races than in other horses. For further details see Supplementary Materials Table S12.

#### 3.9.4. Whip Rule First Breaches in BOBS and Non-BOBS Races

Prima facie, there is not a large difference between the frequency of whip rule breaches for BOBS and Non-BOBS horses in BOBS races, compared with horses in Non-BOBS Races. Further details are provided in Supplementary Materials (see Table S13, and Section S3.9.4. (i) and (ii)).

### 3.10. Fines and Suspensions—2013 Compared to 2016

We compared the number and $ value of fines and the number and length (days) of suspensions imposed in the first four months of 2016 with those in the similar period of 2013 in NSW/ACT. We found there were 15 suspensions (range 4–21 days) and 115 fines (Total $42,650) in the designated 2016 period compared to five suspensions (range 7–10 days) and 36 fines (Total $9400) in the equivalent 2013 period [20].

## 4. Discussion

We identified a need for a peer-reviewed study that characterised whip rule breaches in horse racing from a welfare and regulatory perspective. To achieve this, we used Stewards Reports [20] and Race Diaries [21] from 2013 (and 2016) in a major Australian racing jurisdiction (NSW/ACT). Given the extensive data generated and the number of significant findings, we discuss these under the headings below.

Firstly, however, we provide evidence regarding the nociceptive potential of whip use. Whipping racehorses and its regulation would be irrelevant as animal welfare concerns, if whipping was not potentially painful. McGreevy et al. (2012) reported that 83% of whips strikes viewed in slow motion made a visible indentation [4]. After investigating the mechanical nociceptive thresholds on the dorsal metatarsus in horses, Taylor et al. (2016) stated that “When a noxious mechanical stimulus is applied, pressure distorts the nociceptive nerve endings, activating the nociceptive pathway to the spinal cord” [14]. It was clear these authors were referring to nociception in the horse in general when they explained that the mechanical threshold (MT) is affected by “Numerous additional factors … including the operator, the environment, the anatomical site, the rate of stimulus application and the characteristics of the tissue”. The dorsal aspect of the cannon bone is favoured when measuring MTs as there are minimal anatomical variations between horses, not least because there is very little soft tissue between the skin and periosteum [26].

We argue, therefore, that while the MT for nociception would be expected to vary according to anatomic location, no external part of a normal horse would be insensate. Indeed, the whip is used exactly because the horse feels it. Nociceptive threshold testing in animals is still most commonly used to assess the effects of analgesic drugs in a research setting [14] and studies of potential tissue injury and pain from whipping are scant. Besides the study by McGreevy et al. [4], there appears to be only one article, a conference proceeding from Australia in 1996, which investigated “the potential of whips to injure horses”. This found that “Two factors determine the biophysics of impacts to the body: (1) displacement—when skin and internal structures are stretched/crushed; (2) rate of displacement—reflecting the mechanical “strain-rate sensitive” properties of tissues as pressure waves form during fast impacts” [27].

While the report said “An unequivocal statement on the biomechanics of injury by whips in general is not possible because of the many physiological and physical factors determining tissue damage”, it followed this with “It is, however, possible to develop general principles about a whip’s potential to injure which can be used in a more objective assessment of abuse, thereby allowing the Rules of Racing to be modified to exclude whips with a *greater* potential to injure” (our emphasis). It is disappointing that the authors’ suggestion that “Non-invasive techniques, including thermography and ultrasound … could be useful … to assess whip damage potential and to quantify whip injury after suspected whip abuse” has not, to our knowledge, yet been undertaken.

Notwithstanding this knowledge gap, Taylor et al. (2016) said that while “In animals … the precise sensation experienced is unknown … it is assumed that if a stimulus that produces pain when applied to humans elicits an appropriate response in an animal, this stimulus represents the threshold for pain (Le Bars et al. 2001)” [14]. A 2004 review of the known physiological properties of specialised mechanoreceptors in humans and experimental animals stated “Pain is very often but not always associated with a mechanical stimulus. The mechanical stimulus that causes pain might be an intense one such as pinching of the skin, traumatic injury, or under neuropathic conditions brush or light pressure” [13].

Further, in Australia there is a legal obligation under jurisdictional animal welfare acts to comply with the Australian code for the care and use of animals for scientific purposes 8th edition 2013 (the Code) [28] when research using animals, including horses, is conducted. Section 1.10 states “Animals have a capacity to experience pain and distress, even though they may perceive and respond to circumstances differently from humans. Pain and distress may be difficult to evaluate in animals. Unless there is evidence to the contrary, it must be assumed that procedures and conditions that would cause pain and distress in humans cause pain and distress in animals. Decisions regarding the possible impact of procedures or conditions on an animal’s wellbeing must be made in consideration of an animal’s capacity to experience pain and distress”. Accordingly, we argue that whip use that indents a horse’s skin [4] must be assumed to be painful, unless there is evidence to the contrary, which there is not, and that restrictions on whipping can generally be seen as a step toward the more humane treatment of horses in racing. 

### 4.1. Frequency and Location of Whip Rule Breaches

We found whip rule breaches were recorded by Stewards in NSW/ACT in 2013 in 1% of starts; 6% of races; 31% of race meetings, and at 53% of racetracks. Only a small number of starts with breaches had second breaches in the same race (11%), and over 90% of horses were the only horses in their races with a breach or breaches.

In a study by McGreevy et al. [4] using side-on high speed footage (2000 frames per sec) of 15 race finishes at 2 P NSW race meetings in 2011, 28 whip rule breaches in 9 horses were identified that were not recorded by Stewards. Using the Race Diary [21] to obtain the number of starts in these races, we calculated 6% of these starts had at least one whip rule breach. For the same starts, Stewards recorded a breach frequency of 1% that corresponded to one whip rule breach in a horse that ran tenth and hence was not included in the McGreevy study. Given this study used footage obtained from only one side of the track, and at race finishes, it provides strong evidence that the Stewards under-reported whip rule breaches in the 15 races studied in 2011. As the same surveillance techniques were also used by Stewards in 2013, it is possible that the 1% frequency of whip rule breaches recorded by Stewards for that year, may also underestimate the actual number of whip rule breaches. Of course, it is possible that the breach frequency observed in the small number of races looked at by McGreevy et al. was an anomaly, but it may also indicate the need for faster footage than that currently examined by Stewards (25 frames per sec) [4], which views races head-on. It may also be possible that Stewards focus on the worst offending rider in a race, perhaps because of time pressures, and as such may overlook other riders who are also breaching whip rules. Current surveillance methods should be investigated to ensure these are not failing to detect breaches.

The UK Review found a breach percentage of 0.74 for flat turf racing between 2004 and 2011, which appears slightly higher than the raw 2013 NSW/ACT percentage. However, it needs to be considered that the UK whip rules set a maximum of 15 whip strokes per race [12] (p. 14), whereas that was not the case in 2013 in Australia, nor is it the case now where “In the final 100 m of a race … a rider may use his whip at his discretion” (AR. 137A. (5)(b)).

Following this Review, whip use was further restricted in the UK and is currently capped at 7 strikes per flat race, but penalties are imposed at the discretion of the Stewards [29], as also occurs in Australia. In 2015, the number of UK whip offences, expressed as a percentage of total rides, was 0.61% compared with 1.12% in 2010 [29]. As Racing Australia (RA) does not publish these data, it is not currently possible to say whether the number of whip breaches in NSW/ACT is increasing, decreasing, or static. This shortcoming should be addressed by an industry commitment to reporting, or by mandatory reporting, as part of incremental improvement in racehorse welfare and racing integrity.

Our study found starts at C locations were significantly less likely to result in a breach being recorded than starts at P and M locations, and starts at M locations were significantly more likely to result in a breach being recorded than starts at C and P locations. We were advised there was no difference between the level of surveillance used by Stewards to detect whip breaches at these locations [30] and that Stewards are not attached to individual tracks. Instead, a Stewards panel based in the metropolitan area services M and P race meetings and, in regional centres, Stewards are based in five locations [31]. It is possible, therefore, that reported differences in breach frequencies at C, M and P locations may reflect true differences in the numbers of breaches occurring, or they may reflect behavioural differences in reporting among individual Stewards or between the two pools of Stewards. Further studies are needed to confirm this.

We thought total prize money on offer might incentivise whipping, but our results did not demonstrate this. As Racing NSW describes the BOBS as “the most popular racing incentive scheme in Australia” [32] (p. 30), we also investigated whether it might incentivise whip breaches, but again this was not the case. However, it is possible that the desire to win might have been an incentive, as we demonstrated riders of horses finishing first, second, or third had significantly more whip rule breaches recorded against them than riders of horses finishing in other positions. While our findings support those of Evans and McGreevy that “whippings were associated with superior performance and that there was an association between final placing and the number of whip strikes in the final 200 m section” [3], it should be remembered that in that study, only whip-rule-compliant whip use was included and race footage was not examined by the authors. In this instance, we would want to exclude the possibility that Stewards may tend to focus on the horses running first, second or third, and, as such, possibly miss whip rule breaches by riders of other horses.

### 4.2. Types and Location of Whip Rule Breaches

Our findings also characterise the types and locations of whip rule breaches recorded by Stewards in NSW/ACT in 2013. We now know that the whip rules most commonly recorded as first breaches were forehand whip use on more than five occasions prior to the 100 m mark (Code 17), and using an action that raises the jockey’s arm above shoulder height (Code 8), which accounted for over 45% and 25% of first breaches, respectively. The finding that 70% of first breaches consisted of breaches of only two whip rules deserves further attention, as does the finding that 55% of first breaches consisted of breaches of Code 16 (Forehand whip use in consecutive strides prior to the 100 m mark) and Code 17, which also applies before the 100 m mark.

Possible reasons for these findings include that these breaches may be more easily detected than some others, as Stewards view the race footage head-on [4]; Stewards prioritise these breaches (this does not appear to be the case from the Racing NSW Rider Penalty Guidelines for Whip Rule Breaches (see Appendix A) [33] that were used in 2013, but discretionary biases might occur in practice), or simply these breaches are what might be expected if whip strike frequency and force are perceived to be needed for a horse to win: that is, there is an increased number of whip strikes, and an increased number of more potentially powerful whip strikes with the whip wielded above the jockey’s shoulder. However, McGreevy et al. [4] stated “It is not clear how much, if any, amplification in force that whipping from this height creates. From the perspective of force and therefore pain, the recoil of the whip may be more important than the height from which it descends during its trajectory”. Given we found over a quarter of all first breaches involved whip use when the jockey’s arm is raised above his shoulder height, this question regarding the force of these whip strikes requires clarification.

Importantly, an increase in the number of whip strikes prior to the 100 m mark does not tell us about the actual number of whip strikes used in the final 100 m (except it seems unlikely riders would decrease whip use in this crucial race segment). This contention is supported by Evans and McGreevy [3] who found 98% of horses were whipped in the final 200 m of five sprint races at an M location in NSW, when they were slowing. Evans and McGreevy concluded their data “make whipping tired horses in the name of sport very difficult to justify”. Our data also show whip breaches occurring when horses are likely to be fatigued.

While Whip Rule AR 137A. (3) (Code 6) prohibits excessive, unnecessary, or improper whip use throughout the race, only 3% of first breaches fell into this category. This might suggest such whip breaches do not occur frequently in the critical last 100 m of the race, but this is unlikely given even a cursory examination of race finish footage in the media. One of Australia’s leading sports journalists, Patrick Smith, wrote: “as the rules are now, jockeys will whack their horses non-stop down the straight from the 100 m post…” [17]. The low number of breaches recorded for this whip rule might also reflect problems in detection (as suggested by McGreevy et al. [4]), or simply the Stewards’ discretion.

It is also unclear what constitutes excessive, unnecessary, or improper whip use (AR 137A. (3)) (Code 6), given AR 137A. (4) prohibits whip use (a) forward of horse’s shoulder/vicinity of head (Code 7); (b) that raises arm above jockey’s shoulder height (Code 8); (c) when horse is out of contention (Code 9); (d) when horse is showing no response (Code 10); (e) after passing winning post (Code 11); (f) causing injury to the horse (Code 12); (g) when horse is clearly winning (Code 13); (h) when horse has no reasonable prospect of improving/losing position (Code 14); and (i) in such manner that the seam of the flap is the point of contact with horse, unless rider satisfies Stewards this was neither deliberate nor reckless (Code 15).

Considering that, in 2013, AR 137A. (5)(a) prohibited (i) forehand whip use in consecutive strides prior to the 100 m mark (Code 16) and (ii) forehand whip use on more than five occasions prior to the 100 m mark (Code 17), it appears by default that Code 6 breaches involving whip use in consecutive strides and excessive numbers of whip strikes applied only to the final 100 m of the race. The very low frequency of Code 6 breaches (excessive, unnecessary, or improper whip use) recorded in 2013 provides an impetus to investigate at what stages of races these breaches are reported in, what constitutes these breaches, and in general what percentage of all breaches occur and are recorded in the final 100 m.

Additionally, Code 6 breaches raise the important issue of what Stewards consider to constitute “unnecessary” whipping. This notion logically relies on the concept of “necessary” whipping and there remains a paucity of peer-reviewed evidence to support this, either for safety, or to improve performance [8]. Further, there is a legal argument to consider in relation to what constitutes “unnecessary” under the NSW *Prevention of Cruelty to Animals Act 1979* (POCTA) [34]. Section 5 prohibits cruelty to animals, including racehorses, and Section 4(2) explains “…a reference to an act of cruelty committed upon an animal includes a reference to any act or omission as a consequence of which the animal is unreasonably, unnecessarily or unjustifiably:

(a) beaten, kicked, killed, wounded, pinioned, mutilated, maimed, abused, tormented, tortured, terrified or infuriated,
(b) over-loaded, over-worked, over-driven, over-ridden or over-used…
  or
(d) inflicted with pain”, where “pain” includes suffering and distress (Section 4(1)).

It appears critical this question of whether whipping racehorses could constitute cruelty under the POCTA is considered. Jones et al. [8] discuss “necessary” in relation to animal welfare legislation by using the leading case on this question—Ford v. Wiley, an English High Court decision from 1889 that developed a test to determine when suffering caused to an animal could be deemed to be unnecessary in a particular set of circumstances. It would appear overdue that this be applied to whip use in horse racing in Australia, and particularly in relation to the regulatory outcomes for Code 6 breaches (excessive, unnecessary, or improper whip use).

We also found significant differences between the likelihood of certain whip rule breaches being recorded at the three race locations. Starts at M locations were significantly more likely to result in Code 8 first breaches (Using an action that raises the jockey’s arm above shoulder height) than starts at C locations, and starts at C locations were significantly less likely to result in Code 16 breaches (Forehand whip use in consecutive strides prior to the 100 m mark) than starts at other locations, and significantly more likely to result in Code 17 first breaches (Forehand whip use on more than five occasions prior to the 100 m mark) than starts at M locations.

As discussed in relation to the significant differences we found in the frequency of whip breaches recorded between locations, the significant differences in the types of breaches being recorded at the different locations may reflect true differences in the types of breaches occurring, or perhaps differences in detection and recording amongst individual Stewards or between the two pools of Stewards. Further studies should investigate this.

We found no differences between locations for the recording of Code 9 breaches (Horse whipped when out of contention), which comprised 13% of first breaches. This may reflect our earlier discussion that the desire to win, or possibly not to come last, may lead riders into this whip use, and that these incentives apply at all locations. It is also possible Stewards may be more likely to record a Code 9 breach for a horse that runs last at any location, as it may be more obvious the horse was out of contention than for other non-winning/non-place getter horses. It is worth noting that being out of contention is an holistic evaluation that many observers could arrive at, despite the difficulties in providing a quantitative approach to this rule.

When we examined the types of breaches recorded in horses that ran last, we found most were Code 9, and conversely most Code 9 breaches were recorded in horses that came last. Not surprisingly, horses with these breaches recorded won significantly less money than horses with other breaches in races at all locations.

McGreevy et al. [9] provided insights into Code 9 breaches when they noted in relation to UK and Australian horseracing “…no definition of being “out of contention” is offered by either set of rules and therefore is open to considerable interpretation”. Evans and McGreevy [3] further highlighted the conundrum facing a jockey to both “ride his horse out (i.e., ensures the horse gave of its best) to the end…” in accordance with AR 137. (b). It appears that in 2013 these issues had not yet been sufficiently clarified, and this remains the case currently. 

In interpreting our data on second breaches, it must be remembered that while the level of surveillance may be the same at all locations, Stewards may use their discretion to order first and second breaches differently at the three locations, or there may be no difference in the importance of what breach is recorded first. Notwithstanding this, we are able to say that where two breaches were recorded in one start, over 75% involved the same two whip rules (Codes 16 and 17) and hence occurred prior to the 100 m mark. Attempts to clarify what determined the order of two breaches recorded in a start and whether this affected what penalties were imposed, were answered simply as “both penalised” [30]. This highlights the need for a transparent prosecution policy or penalty guidelines in Australia, as now provided in the UK by the British Horseracing Authority (BHA) [35]. Likewise, our examination of Stewards Reports showed a need for greater consistency in how information is recorded between racetracks.

Several whip rules had no breaches recorded. In some cases this may reflect a lack of clarity of the rules, or difficulties in detection, rather than indicating a 100% compliance rate (though that is probably the case with AR 137A. (1) and (2), which pertain to the correct, padded whip being used).

For example, in the same study that revealed indentations in the skin of horses in over 80% of whip strikes in 15 race finishes at two P NSW race meetings in 2011, McGreevy et al. [4] raised concerns “the seam rule” (Code 15) “is virtually impossible to police, even using significantly more detailed footage than is usually reviewed by racing Stewards, and its inclusion is therefore futile”. The authors noted “the number of times breaches of the seam contact rule have ever been prosecuted since the current rules were established is negligible”. Our finding that there were no breaches of this whip rule recorded by Stewards in 2013 supports McGreevy et al.’s contention, and further questions the adequacy of monitoring of this rule by Stewards, and the validity of the rule itself.

Similarly, it appears likely that current methodology employed by Stewards is not sufficiently sensitive to detect tissue injury caused to horses from whipping, except in extreme cases where a weal (or welt) is visible to the naked eye. This may explain why there were no recorded breaches of the “injury” whip rule (Code 12) in 2013, despite evidence that whip use in racing frequently indents the skin of horses and as such may injure it [4,13,14,26,27].

Currently, horses that have raced are examined by industry veterinarians before they are removed from the racecourse. We argue this may fail to expose inflammatory processes and, hence, ensure whip rules prohibiting injury to the horse are complied with. Where feasible, the examination of horses the day after racing with advanced tools, such as thermography, would help clarify the extent of whip-related injuries.

In their 2012 study, McGreevy et al. also showed that the unpadded section of the whip made contact with the skin of the horse in 64% of impacts, and challenged the notion padded whips (as prescribed in AR 137A. (1) and (2)) prevent injury to the horse. The reason proponed was the padding is held onto the shaft of the whip with unpadded binding that strikes the horse whenever the padded section does [4].

Our study considered whip use that was recorded by Stewards as breaching the whip rules and, hence, in all likelihood analysed only a small proportion of whip strikes overall. This can be easily appreciated when you consider AR 137A. (5)(b) allows the rider to use “his whip at his discretion” in the final 100 m of every race, where the rules restricting the number of strikes and whip use in consecutive strides do not apply. The Stewards reports show therefore that the minimum number of horses whipped in NSW/ACT in 2013 was 384 (including 37 horses in which second breaches were recorded). In light of McGreevy’s findings [4], our contention is that these horses would also have been struck with the unpadded part of the whip in many of these impacts. Indeed, we argue that this would also have been the case in many of the whip strikes that did not breach the whip rules and hence were not the focus of our study.

Likewise, another of McGreevy et al.’s findings [4] was that the horse’s flank (abdomen) is “twice as likely to be struck with the entirety of the whip than is the hindleg”. There is no evidence to suggest this unintended whip action was not still occurring in 2013. As McGreevy et al. highlighted, although this area of the horse is regarded as being “particularly sensitive to tactile stimulation”, and UK whip rules restrict whip use to the quarters of the horse, no similar safeguard is provided in Australia. This is surprising given Australia is one of 49 countries that signed the International Agreement on Breeding, Racing and Wagering [36], which prohibits the use of the whip on the flank. It is unclear why flank strikes are not reported under Whip Rule AR 137A. (3) (Code 6), which prohibits improper whip use. It may be bilateral race footage is needed before any assurance about proper whip use can be provided.

Similarly, the International Agreement’s prohibition on the use of the whip with excessive force cannot be effectively policed in the absence of whips that detect force. One such electronic whip, which counts whip strikes and their force, is currently being advertised [37]. While this type of device might work to limit the force of whip impacts, it would be unlikely to prevent possible injury resulting from repeated strikes to the same area, or take into account differences in pain thresholds and susceptibilities to injury in individual horses.

Further, as the Stewards did not record any breaches in 2013 involving whip use when the horse was showing no response (Code 10), this implies firstly that the Stewards are able to detect such responses, even when horses are slowing; and secondly, that all whipped horses responded to the Stewards’ satisfaction. The training and evidence-base required to produce and assess this skill should be critically examined.

These problems in the detection and enforcement of whip rule breaches raise important issues about Australian whip rules. The need for laws (or rules) to be clear is the first Rule of Law Principle identified by the Law Council of Australia [38] (p. 1). This is not only so those who are regulated understand what they cannot do, but also to prevent arbitrary enforcement and erosion of respect for the law. Sophocles warned “What you cannot enforce, do not command”, and this is relevant to the integrity issues facing racing today. Given the industry is currently permitted to self-regulate, it is even more important that the AR are clear, enforceable, and enforced fairly and consistently. If breaches of rules are hard to quantify, then the rules need to be reworded to allow detection without quantification so that a binary (yes/no) outcome can be recorded.

### 4.3. Race Length, Location and Whip Rule Breached

We found the length of races at M locations varied significantly with some whip rule breaches. It may be at these tracks, riders wield the whip above the shoulder more (Code 8) and also whip when out of contention (Code 9) more in shorter races, while in longer races there is more opportunity to use additional whip strokes (hence, more Code 17 breaches). So, in shorter races we might be seeing “Fast and Furious” whip use, in which the rider uses whip strokes that arguably have greater force, while also having less time to decide if the horse is out of contention or, indeed, responding.

When we examined race length according to race type, and considered first and second breaches equally, we found most breaches were recorded in sprint races (which may support the “Fast and Furious” hypothesis), with less than half as many breaches recorded in mile and middle distance races. Not surprisingly, Code 16 and 17 breaches accounted for the majority of breaches recorded in all race types, and again reflects the strong recording of these whip breaches that occur prior to the 100 m mark.

### 4.4. Rider Gender, Type and Whip Breaches

It was outside the scope of this study to collate the raw data for the total number of jockeys and apprentices, and total number of male versus female riders, in all starts in 2013. Hence, we are unable to say which gender/type of rider is more likely to have whip rule breaches recorded. However, we noticed a different pattern of breaches between these groups.

Nearly six times as many male riders had starts with breaches recorded compared to female riders. However, as only the gender of riders with breaches recorded was known, the pertinence of this difference is unclear. Certainly, once one breach was recorded, there was no association between gender and the recording of a second breach. However, we did find that of riders with breaches recorded, female riders were significantly underrepresented for Code 17 breaches (Forehand whip use on more than five occasions prior to 100 m mark), and overrepresented for Code 9 breaches (Whip use when the horse is out of contention). These are interesting findings regarding possible gender effects, but again require further investigation.

Aligning with a previous study of Australian jockeys and apprentices [39], our results also showed that the type of whip rule recorded as breached depended on jockey status. Among riders with breaches, apprentices were significantly more likely than jockeys to have breaches of Code 9 (Whip use when the horse is out of contention) recorded at C and P locations. Given we also found no differences between locations for Code 9 breaches recorded overall, it is possible that, at M locations, jockeys may also be breaching this whip rule at the same frequency as apprentices. This may reflect a greater desire by all riders at the high profile M races to win, or not to run last, or again reflect differences relating to individual Stewards or between the two pools of Stewards.

While we acknowledge that only 1% of starts had whip rule breaches recorded in NSW/ACT in 2013, it is still concerning that just over 50% of riders responsible for breaches were repeat offenders (that is, they had more than one whip rule breach recorded in 2013, including second breaches in a start). Nearly twice as many male riders than female riders were in this category. Of these, there were nearly twice as many jockeys (of which just over half were repeat offenders) than apprentices (of which just under half were repeat offenders). The number of repeat offenders in all categories is troubling, especially apprentices as they are still in training and should be “on probation”. It would appear, in 2013 at least, there were insufficient deterrents to repeat offending, and possibly a lack of effective education regarding whip rules for apprentices. In a pilot study, McGreevy et al. found 15 of 24 jockeys said trainers and owners were sources of pressure to use the whip [40]. This and other possible reasons should be further investigated in relation to gender, type of rider, type of whip rule breach, and location.

### 4.5. Breach Outcomes

Overall, breach outcomes or penalties were low in our view. Reprimands were the most common outcome for first and second breaches, with less than a third of first breaches resulting in fines, and even then the total was a modest $25,600 (median value $200). Further, all Code 6 first breaches (Excessive, unnecessary, or improper whip use) resulted in cautions or reprimands only.

The four most commonly recorded first breaches had variable outcomes. For example, Code 8 first breaches (Whip use that raises arm above jockey’s shoulder height) had the highest fines imposed overall (2 × $800), while fines were rare for Code 9 breaches (Whip use when the horse is out of contention), with the highest fine imposed being $300.

In 2013, Stewards imposed only 13 suspensions for whip rule breaches, which ranged from 5 to 14 days, and all resulted from breaches of Codes 16 and 17. No cautions were issued for Code 16 breaches, and only one was issued for a Code 17 breach, which might imply Stewards regard these more seriously than other whip breaches.

We sought the Stewards’ “prosecution policy or similar” that helped determine the penalties imposed for whip rule breaches in 2013, as this was not available on Racing NSW’s website. The resultant advice was there “is only a guideline” (see Appendix A) [33], and this related to Code 16 and 17 whip breaches only. Although not stated in the Guidelines, suspensions appear to be a serious outcome and are not suggested as outcomes for breaches of Code 16 (Forehand whip use in consecutive strides prior to 100 m mark) or Code 17 (Forehand whip use on more than five occasions prior to the 100 m mark), until the sixth offence for both 4–5 whip strikes in consecutive strides (Code 16) and 4–5 whip strikes in excess of the five allowed under Code 17; for the second offence for six or more whip strikes in consecutive strides (Code 16), and for six or more whip strikes in excess of the five allowed under Code 17.

While Guidelines were provided to us for only these two whip rules, it may be possible Stewards use additional guidelines. Penalties are also always at the Stewards’ discretion. Nonetheless, it is telling that in 2013 suspensions were imposed only for Code 16 and 17 breaches, and thus were for whip breaches that took place prior to the 100 m mark.

Although Stewards can impose loss of winnings and earnings on riders breaching whip rules (AR 196. (2)), this did not occur [41]. Overall, the penalties imposed for whip rule breaches in 2013 appear low, with the majority not involving fines or suspensions, and when these were imposed they appeared insufficient to act as serious deterrents (as seen in, what the current authors consider to be, the high number of repeat offenders). 

### 4.6. Riders with the Highest Number of Whip Breaches

Our aim here was to provide a preliminary insight into the type of rider whose whip use attracted the Stewards’ attention in 2013. Of 15 riders with the highest numbers of breaches, most were jockeys and male, the number of starts with one or more breaches ranged from six to 13, and nearly 75% of these riders had starts with second breaches.

There were also 13 race meetings where the same riders had breaches in two races, with one rider having breaches in two races at two different race meetings. Four of the riders were apprentices (two males, two females) and seven were jockeys among the 15 riders with the highest number of breaches overall and were male.

When we considered the five riders with the four highest numbers of starts with breaches, their fines as a percentage of their 4.95% share of the total prize money won ranged from 11% to 407%. So, for some riders, the fines for whip breaches appear to have considerable impact on their share of the prize money, but we do not know whether this is compensated for in other races in which they do not breach the whip rules, or are not recorded as doing so. It is interesting that the jockey with the highest number of starts with breaches and the highest fines overall ($4300), rode horses with winnings of $238,335, of which his share was $11,797. For this jockey, 36% of his winnings were lost as fines. It may be that what riders lose in individual races in $ terms from whip rule breaches, is out-weighed by improvements to the riders’ winning statistics and, hence, translate to more and higher quality race work.

### 4.7. Possible Whip Breach Deterrents

While over a million dollars in total prize money was won by horses in starts with breaches, the median percentage of a fine compared to the prize money won in an individual case was only 14%, though this ranged widely from almost zero % (for a horse that won $105,000 but the rider was fined $200) to eight cases in which the fine exceeded the prize money won.

Overall, fines for whip rule breaches represented only 2% of the total prize money won by these horses in 2013. Similarly, we found fines represented almost zero % of total prize money on offer in races with breaches, and like prize money won, was unlikely to act as a meaningful deterrent.

### 4.8. The 2015 Whip Reforms and the Current Situation

In 2013, backhand whip use, where the whip is held like a ski pole, as opposed to forehand whip use, where the whip is gripped like a tennis racquet [9], was exempted from AR 137A. (5)(i) and (ii) (Codes 16 and 17). McGreevy et al. opined in 2012 [4] that it was possible “that the rules have inadvertently encouraged jockeys to use the backhand rather than the forehand actions to avoid being penalised”. The veracity of this concern was reinforced by whip rule reforms enacted on 1 December 2015 by RA. In a letter to jockeys, John Messara AM (Chairman, RA) said:

“Since the introduction of the 2009 reforms, riders have become increasingly proficient at whipping in the backhand manner. Many backhand strikes can be equated in force with forehand strikes.

Australia’s current whip rules are not best international practice when benchmarked against other major racing jurisdictions. Racing is accountable for the highest standards of animal welfare, in line with community standards. Community standards require a new regime of whip usage which is tailored towards principles of horsemanship rather than punishment. Australia has an international reputation for leading the world on animal welfare issues and we want to maintain it”.

[42]

An RA Media Release on 23 October 2015 further explained:

“In respect of Australian Rule of Racing AR. 137A., the Board has decided:

1. To remove the distinction between forehand and backhand whip strikes so that there is a limit of five forehand or backhand whip strikes prior to the 100 m.
2. To introduce stronger penalties for whip offences including greater emphasis on suspensions for serious breaches and for breaches in Group and Listed races.

The rule changes are largely an extension of the whip reforms of 2009. The changes to the whip rules in 2009 introduced limits on the number and manner of whip strikes which in conjunction with a padded whip has ensured the welfare of the horse.

However, too great a reliance on the backhand application of the whip has developed in response to the limits imposed on the forehand application. After careful consideration, the Board decided that backhand strikes should be treated in the same way as forehand strikes so as to leave no room for misinterpretation of the rules against excessive use”.

[43]

Unfortunately, the 2015 reforms do little to assist interpretation of what constitutes excessive/unnecessary/improper whip use (Code 6-AR 137A. (3)), including in the final 100 m. While whip rule reforms were much needed, it is also unfortunate the reform process was not evidence-based, more transparent, and inclusive to stakeholders including scientists, welfare groups, and the community. What the reforms tell us, though, is that our 2013 data very much underestimated the true extent of whip use prior to the 100 m mark, because backhand strikes were excluded. This is concerning, especially as McGreevy et al. [9] found that while the forehand versus backhand action does not influence the force on impact when using the non-dominant hand, when using the dominant hand, the jockeys in the study (using a model horse), struck with more force in the backhand.

We briefly reviewed the fines and suspensions imposed in the first four months of 2016 with those in the similar 2013 period in NSW/ACT, given RA’s 2015 pledge to introduce stronger penalties for whip offences. We found there were four suspensions (range 7–10 days) and 35 fines totalling $9400 in 2013, and in the same period in 2016, there were 15 suspensions (range 4–21 days) and 115 fines totalling $42,650 [20]. It is beyond the scope of the current study to consider this in detail, but given the increase in the number and $ value of fines, and the number and length of suspensions, these data should be examined further. While RA has been very vocal in justifying the higher penalties it imposed at this year’s Autumn Racing Carnival [44], industry reporter Adrian Dunn wrote that for this period “NSW—which has held more meetings than any other state—is not facing the same issue as Victoria and Queensland. While NSW has issued more whip-infringement suspensions (12) than Victoria, it has delivered only 99 fines—less than half that of Victoria” [45].

On 24 June 2016, RA announced a review of the new whip rules since their introduction last December [46]. Hopefully, this will examine the reasons for the cited jurisdictional differences above, as well as evaluate whether the reforms have achieved the intended outcomes, which presumably include rectifying the assessment that Australia’s whip rules, prior to the reforms, were “not best international practice” [42].

Based on our findings from 2013, when the whip rules were the same as they were immediately prior to the December 2015 reforms, there is a case to argue that a review of all the whip rules is warranted, and not just those pertaining to whip use prior to the 100 m mark. Any review should be science-based and include independent comparison with the UK where, unlike Australia, the total number of whip strikes is restricted. Nonetheless, the increased number of fines and suspensions in the 2016 period may indicate a step toward better regulation of whip use and welfare, even if it is just that backhand strikes are now being penalised.

Knight and Hamilton [47] stated in relation to the UK Review that “The conclusion that the whip contributes to rider safety has important implications under Australian Work Health and Safety legislation and therefore affects the ability of racing authorities to change the rules relating to whip use”. We argue this makes it all the more important that studies that direct policy and regulatory change are based on peer-reviewed studies using race data, much of which are already being collected by industry. Some whip use information is not currently available, but must be if properly informed decisions are to be made about the use of whips in racing. In 2012, McGreevy and Ralston raised the case “for publication of the numbers of official whip strikes per horse per race, a move that is likely to increase transparency in the activity of stewards” [39].

Further, in her paper “Sustainability, thoroughbred racing and the need for change”, Bergmann wrote: “Improving transparency and regulation is important and can improve welfare outcomes, if transparency and regulation go beyond the aim of protecting the integrity of the race and shift the focus on protecting the horse” [10]. Similarly, in their paper “Science alone is not always enough; the importance of ethical assessment for a more comprehensive view of equine welfare” [11], Heleski and Anthony quote Grandin’s saying that it is easy for “bad to become normal” [48].

## 5. Conclusions

This multi-disciplinary study provides the first peer-reviewed characterisation of whip rule breaches recorded by Stewards and their regulatory outcomes in horseracing. This was achieved using Stewards Reports and Race Diaries, and improves understanding of whip use and compliance in a major Australian racing jurisdiction. Importantly, it is a step toward rectifying the current knowledge gap in this increasingly contentious area.

Whip use in horse racing is an important animal welfare issue. While there is a lack of peer-reviewed evidence that specifically demonstrates that whipping horses causes pain, we argue there is sufficient evidence now and a moral and legal imperative to assume that, in the absence of evidence to the contrary, whipping is potentially painful. As such, restrictions on whip use have the real potential to improve the welfare of horses in racing.

Not only is there growing community concern about the welfare of horses being whipped in races, whipping, and its regulation, is also a racing integrity issue. Our finding that there was a significantly higher frequency of recorded breaches by riders of horses that finished first, second, or third than by riders of horses that finished in other positions, suggests a desire to win may incentivise whip rule breaches and potentially affect race and betting outcomes. Likewise our finding that there was a significantly higher frequency of recorded breaches at M than at C or P locations may reflect the desire to win may be particularly high at the more prestigious ‘city’ race tracks. This preliminary study raises a number of questions that need to be answered including whether the number of breaches recorded by Stewards aligns with the actual number of whip rule breaches at all locations, and what Steward and surveillance-related factors might affect whether a breach is recorded or not.

It is also concerning from a regulatory point of view that a small number of types of whip breach predominated in 2013, with no breaches recorded for several whip rules, despite evidence to suggest some of these may be breached regularly. Our study showed that over half of all first breaches recorded occurred prior to the 100 m mark and involved two whip rules. Unfortunately, with the exception of these, the Stewards reports do not specify at which race stage whip breaches occur. Hence it is not clear how many breaches are recorded in the crucial final 100 m of races. Regulatory outcomes or penalties were generally low and, in our view, insufficient to act as deterrents. Our findings strongly suggest that the whip rules, their surveillance and recording, and the penalties imposed warrant urgent and independent review.

Likewise, the associations our study reveals between recorded whip rule breaches, racing data variables, and outcomes provide a strong case for such analyses to be undertaken annually to inform the evidence-base for policy, education, and regulatory change, including breaches and penalties.

Indeed, this study raises several issues pertinent to horse welfare and racing integrity that warrant further investigation. We hope it will also lead to more evidence-based whip rules that meet growing community expectations that horses in racing will be treated humanely.

## Figures and Tables

**Table 1:** Whip rules breached in 348 first breaches.

Breach Code	Whip Rules Breached	Whip Rule Description	Number (out of 348)	Percentage
1	AR 137A. (1)(a)	Only padded whips of a design and specifications approved by a panel appointed by the ARB may be carried in races or official trials;	0	0
2	(1)(b)	Every such whip must be in satisfactory condition and not modified in any way;	0	0
3	(1)(c)	Stewards may confiscate any whip not in a satisfactory condition or modified;	0	0
4	(1)(d)	Any rider found guilty of above may be penalised. Provided the master and/or other person in charge of an apprentice jockey at the relevant time may also be penalised unless they satisfy Stewards all proper care to ensure the apprentice complied with the rule was taken;	0	0
5	AR 137A. (2)	Only whips of a design and specifications approved by ARB may be carried in track work;	0	0
6	AR 137A. (3)	Excessive/unnecessary/improper whip use;	10	2.87
7	AR 137A. (4)(a)	Whip use forward of horse’s shoulder/vicinity of head;	6	1.72
8	(4)(b)	Whip use that raises arm above jockey’s shoulder height;	87	25.00
9	(4)(c)	Whip use when horse is out of contention;	44	12.64
10	(4)(d)	Whip use when horse is showing no response;	0	0
11	(4)(e)	Whip use after passing winning post;	0	0
12	(4)(f)	Whip use causing injury to horse;	0	0
13	(4)(g)	Whip use when horse is clearly winning;	1	0.29
14	(4)(h)	Whip use when horse has no reasonable prospect of improving/losing position;	1	0.29
15	(4)(i)	Whip use in such manner that the seam of the flap is the point of contact with horse, unless rider satisfies Stewards this was neither deliberate nor reckless;	0	0
16	AR 137A. (5)(a)(i)	Forehand whip use * in consecutive strides prior to 100 m mark;	33	9.48
17	(5)(a)(ii)	Forehand whip use * on more than 5 occasions prior to 100 m mark;	157	45.11
18	(5)(a)(iii)	Rider may at his discretion use whip with slapping motion down horse’s shoulder, with whip hand remaining on reins, or alternatively in a backhand manner;	0	0
19	(5)(b)	In the final 100 m a rider may use whip at his discretion;	0	0
20	AR 137A. (7)(a)	Any trainer, owner or authorised agent must not give instructions to a rider regarding the use of the whip, which, if carried out, might result in a breach of this rule;	0	0
21	(7)(b)	No person may offer inducements to a rider to use the whip in such a way that, if carried out, might result in a breach of this rule;	0	0
22	AR 137A. (8)	Any person who fails to comply with any provisions of this rule is guilty of an offence;	0	0
23	AR 137A. (9)	An owner or authorised representative, trainer, rider or Steward may lodge an objection against the placing of a horse where the rider contravenes AR 137A. (3) or (5);	0	0
24	Unspecified whip use	Whip Rule breach not specified by Stewards.	9	2.59

The wording of the whip rules has been simplified for the purposes of this article (for exact wording, see Rules of Racing [23]; * Whip rules were amended 1 December 2015 to include backhand whip strikes as well as existing forehand restrictions; ARB—Australian Racing Board (now Racing Australia).

**Table 2:** Whip rules breached in 348 first breaches classified by Country, Metropolitan, and Provincial location.

Breach Code	Country Number out of 200 (%)		Metropolitan Number out of 77 (%)		Provincial Number out of 71 (%)	
6	7	(3.50%)	1	(1.30%)	2	(2.82%)
7	3	(1.50%)	2	(2.60%)	1	(1.41%)
8	32	(16.00%)	40	(51.95%)	15	(21.13%)
9	30	(15.00%)	4	(5.19%)	10	(14.08%)
13	1	(0.50%)	0	(0.00%)	0	(0.00%)
14	0	(0.00%)	0	(0.00%)	1	(1.41%)
16	11	(5.50%)	10	(12.99%)	12	(16.90%)
17	107	(53.50%)	20	(25.97%)	30	(42.25%)
24	9	(4.50%)	0	(0.00%)	0	(0.00%)

See Table 1 for breach code key.

**Table 3:** Outcomes of first and second breaches of whip rules.

Outcome	First Breach (*n*)	Second Breach (*n*)
$100	12	0
$200	61	1
$300	16	0
$400	8	0
$500	4	0
$600	1	0
$800	2	0
Not Known	8	0
Caution	56	2
No Outcome	1	0
Reprimand	170	25
Suspension	9	4
Conviction Recorded	0	5
Total	348	37

**Table 4:** Outcomes of first breaches classified by whip rule breached.

First Breach Code	C	R	100	200	300	400	500	600	800	S	NO	NK
6	6	4	0	0	0	0	0	0	0	0	0	0
7	4	0	0	2	0	0	0	0	0	0	0	0
8	28	32	6	9	1	6	2	1	2	0	0	0
9	16	21	5	1	1	0	0	0	0	0	0	0
13	1	0	0	0	0	0	0	0	0	0	0	0
14	0	1	0	0	0	0	0	0	0	0	0	0
16	0	21	0	7	3	0	0	0	0	2	0	0
17	1	91	1	42	11	2	2	0	0	7	0	0
24	0	0	0	0	0	0	0	0	0	0	1	8

C—Caution; R—Reprimand; 100–800—$ Fine; S—Suspension; NO—No Outcome; NK—Not Known; Code 6—Excessive/unnecessary/improper whip use; Code 7—Whip use forward of horse’s shoulder/vicinity of head; Code 8—Whip use that raises arm above jockey’s shoulder height; Code 9—Whip use when horse is out of contention; Code 13—Whip use when horse is clearly winning; Code 14—Whip use when horse has no reasonable prospect of improving/losing position; Code 16—Forehand whip use * in consecutive strides prior to 100 m mark; Code 17—Forehand whip use * on more than 5 occasions prior to 100 m mark; Code 24—Whip Rule breach not specified by Stewards; * Whip rules were amended 1 December 2015 to include backhand whip strikes as well as existing forehand restrictions.

**Table 5:** Length of races with breaches by type of race and whip rule breached.

Breach Code	Sprint ≤1400 m (*n*)	Mile 1406–1750 m (*n*)	Middle 1800–2400 m (*n*)	Total (*n*)
8	55	24	13	92 (23. 90%)
9	29	11	4	44 (11.43%)
16	33	10	11	54 (14.03%)
17	84	42	42	168 (43.64%)
Other	18	6	3	27 (7.01%)
Total	219 (56.88%)	93 (24.16%)	73 (18.96%)	385

First and second breaches are included for a total number of 385 (348 first breaches + 37 second breaches); Code 8—Whip use that raises arm above jockey’s shoulder height; Code 9—Whip use when horse is out of contention; Code 16—Forehand whip use * in consecutive strides prior to 100 m mark; Code 17—Forehand whip use * on more than 5 occasions prior to 100 m mark; Other—see Table 1 for other breach codes; * Whip rules were amended 1 December 2015 to include backhand whip strikes as well as existing forehand restrictions.

**Table 6:** Fines imposed and prize money won by riders (*n* = 5) with the most breaches in 2013.

Rider Code	Number of Starts with Breaches	Number of Starts with Breaches When Fine Was Imposed	Whip Rule Fines $	Prize Money Won by Horse $	4.95% Rider’s Share of Prize Money Won $	Fine as % Prize Money Won	Fine as % Rider’s 4.95% Share of Prize Money Won
A	13	13	4300	238,335	11,797.58	1.80	36.45
B	12	8	2100	66,230	3278.39	3.17	64.06
C	12	4	1000	178,425	8832.00	0.56	11.3
D	10	2	600	2975	147.26	20.17	407.44
E	9	7	1700	9580	474.21	17.7	358.49

Rider code—each letter represents the name of a different rider and is the same as designated in Supplementary Materials Tables S8 and S9.

## Supplementary Materials

The following are available online at www.mdpi.com/2076-2615/7/1/4/s1. Table S1: Details of tracks, race meetings, races, races with breaches, starts, and starts with breaches for Country, Metropolitan, and Provincial locations, Table S2: Whip rules breached in second breaches classified by Country, Metropolitan, and Provincial location, Table S3: Fines resulting from first breaches, Table S4: Outcomes of second breaches classified by whip rule breached, Table S5: Total prize money on offer in races with breaches by location, Table S6: Length of races with breaches, Table S7: Riders with breaches (including repeat offenders), Table S8: Riders with the highest numbers of breaches, Table S9: Riders breaching whip rules in two races at the same race meeting, Table S10: BOBS and Non-BOBS races classified by Country, Metropolitan, or Provincial location, Table S11: Percentage of BOBS horses in starts classified by Country, Metropolitan or Provincial location, Table S12: BOBS status of 339 horses with breaches classified by Country, Metropolitan or Provincial location, Table S13: Whip rules breached in first breaches in BOBS and Non-BOBS races.

## Abbreviations

The following abbreviations are used in this manuscript:

ACT	Australian Capital Territory
AR	Australian Rules of Racing
ARB	Australian Racing Board
BHA	British Horseracing Authority
BOBS	Breeder Owner Bonus Scheme
C	Country
LR	Local Rules of Racing
M	Metropolitan
NSW	New South Wales
P	Provincial
RA	Racing Australia
$	AUS dollar

## Appendix A

**Racing NSW**

**Rider Penalty Guidelines for Whip Rule breaches **

(To be used as a guide only with all circumstances of the breach to be considered, penalties to remain at the discretion of the Stewards)

**Table:** **400–100** **metres Successive strokes within the 5 permitted strokes AR 137A (5)(a)(i)**

Offence	Successive (2)	Successive (3)	Successive (4–5)	Successive (6+)
1st	Reprimand	Reprimand	$200	$500
2nd	Reprimand	Reprimand	$300	Suspension up to 1 week
3rd	Reprimand	$200	$400	Suspension up to 1 week
4th	$200	$200	$500	Suspension up to 2 weeks
5th	$200	$200	$600	Suspension up to 2 weeks
6th	$200	$300	Suspension up to 1 week	Suspensions up to 3 weeks
7th	$300	$300	Suspension up to 1 week	Suspensions up to 3 weeks
8th	$300	$300	Suspension up to 2 weeks	Suspension up to 4 weeks
9th	$300	$400	Suspension up to 2 weeks	Suspension up to 4 weeks
10th	$400	$400	Fine & Suspension 2 weeks +	Suspension 4 weeks +

Please note number of successive strokes includes total strokes ie 3 = 3 in successive.

**Table:** **400–100** **Metres Additional Strokes in excess of 5 strokes AR 137(A) (5)(a)(ii)**

Offence	1 Additional	2 Additional	3 Additional	4–5 Additional	6 + Additional
1	Reprimand	Reprimand	Reprimand	$200	Suspension up to 1 week
2	Reprimand	Reprimand	Reprimand	$300	Suspension 1 week +
3	Reprimand	Reprimand	$200	$400	Suspension up to 2 weeks
4	Reprimand	$200	$200	$500	Suspension 2 weeks +
5	$200	$200	$200	$600	Suspension up to 3 weeks
6	$200	$200	$300	Suspension up to 1 week	Suspension 3 weeks +
7	$200	$300	$300	Suspension up to 1 week	Suspension up to 4 weeks
8	$200	$300	$300	Suspension up to 2 weeks	Suspension 4 weeks +
9	$300	$300	$400	Suspension up to 2 weeks	Suspension 4 weeks +
10	$300	$400	$400	Fine & Suspension 2 weeks +	Suspension 4 weeks +

For repeated offences involving 6 or more additional or successive strides the penalty may include a fine or the equivalent to the riders percentage of prize money; As a deterrent breaches in Group and Feature races may attract a heavier penalty; If a rider commits a minor breach of both rules, only one, penalty shall apply, that being the greater penalty provided for in the two respective tables i.e., a 7th time offender using one additional stroke of the whip, including one successive application will be eligible to be fined $300 in total; A riders record refreshes after 400 rides or 12 calendar months; Percentage of Prize money means the riders percentage is expressed as a maximum figure for penalty; A Riders breach of the whip rules prior to the 26th of September 2009 are not to be taken into account when assessing penalty.

## References

[1] Jones B., McGreevy P.D. (2010). Ethical equitation: Applying a cost-benefit approach. J. Vet. Behav..

[2] McGreevy P.D., Oddie C. (2011). Holding the whip hand—A note on the distribution of jockeys’ whip hand preferences in Australian thoroughbred racing. J. Vet. Behav..

[3] Evans D., McGreevy P. (2011). An investigation of racing performance and whip use by jockeys in thoroughbred races. PLoS ONE.

[4] McGreevy P.D., Corken R.A., Salvin H., Black C.M. (2012). Whip use by jockeys in a sample of Australian thoroughbred races—An observational study. PLoS ONE.

[5] Graham R., McManus P. (2016). Changing human-animal relationships in sport: An analysis of the UK and Australian horse racing whips debates. Animals.

[6] Horsetalk.co.nz Ban on Whip Use Would Be Positive for Racing, Suggests John Francome. http://horsetalk.co.nz/2015/11/12/ban-whip-use-positive-racing-francome/#axzz40UAtJE5b.

[7] McLean A.N., McGreevy P.D. (2010). Ethical equitation: Capping the price horses pay for human glory. J. Vet. Behav..

[8] Jones B., Goodfellow J., Yeates J., McGreevy P. (2015). A critical analysis of the British Horseracing Authority’s review of the use of the whip in horseracing. Animals.

[9] McGreevy P.D., Hawson L.A., Salvin H., McLean A.N. (2013). A note on the force of whip impacts delivered by jockeys using forehand and backhand strikes. J. Vet. Behav..

[10] Bergmann I. (2015). Sustainability, thoroughbred racing and the need for change. Pferdeheilkunde.

[11] Heleski C.R., Anthony R. (2012). Science alone is not always enough: The importance of ethical assessment for a more comprehensive view of equine welfare. J. Vet. Behav..

[12] British Horseracing Authority (2011). Responsible Regulation: A Review of the Use of the Whip in Horseracing. http://www.britishhorseracing.com/wp-content/uploads/2014/03/WhipReview.pdf.

[13] Lewin G.R., Moshourab R. (2004). Mechanosensation and pain. J. Neurobiol..

[14] Taylor P.M., Crosignani N., Lopes C., Rosa A.C., Luna S.P.L., Puoli Filho J.N.P. (2016). Mechanical nociceptive thresholds using four probe configurations in horses. Vet. Anaesth. Analg..

[15] SBS News (2015). Greens back RSPCA Call to Ban Horse Whips. http://www.sbs.com.au/news/article/2012/03/21/greens-back-rspca-call-ban-horse-whips.

[16] The Coalition for the Protection of Racehorses Proposal for the Phasing out of the Whip in Australian Thoroughbred Racing March 2015. https://www.horseracingkills.com/campaigns/the-whip/proposal-for-phasing-out-the-whip/.

[17] The Australian As Public Turns against Racing Codes, Whip Goes Under Review. http://www.theaustralian.com.au/sport/opinion/patrick-smith/as-public-turns-against-racing-codes-whip-goes-under-review/news-story/f3cc57b7ae1402a3a8874623a20d233c.

[18] RSPCA Policy C05 Horse Racing. http://kb.rspca.org.au/RSPCA-Policy-C05-Horse-Racing_642.html.

[19] British Horseracing Authority (2011). Responsible Regulation: A Review of the Use of the Whip in Horseracing. http://www.britishhorseracing.com/wp-content/uploads/2014/03/WhipReview.pdf.

[20] Racing NSW Stewards Reports. http://new.racingnsw.com.au/default.aspx?s=race-diary-stewards&filter=Stewards.

[21] Racing NSW Race Diary. http://racing.racingnsw.com.au/FreeFields/Calendar_Meetings.aspx?State=NSW.

[22] Racing NSW BOBS General Information. http://www.racingnsw.com.au/default.aspx?s=general-information-bobs.

[23] Racing NSW Rules of Racing of Racing NSW. http://www.racingnsw.com.au/site/_content/document/00000401-source.pdf.

[24] R Core Team (2016). R: A Language and Environment for Statistical Computing.

[25] Murrihy R. (2016). Chief Steward NSW Racing, Sydney, NSW, Australia.

[26] Love E.J., Murrell J., Whay H.R. (2011). Thermal and mechanical nociceptive threshold testing in horses: A review. Vet. Anaesth. Analg..

[27] Mills P.C., Higgins A.J. Investigation of the potential of whips to injure horses. Proceedings of the 11th International Conference of Racing Analysts and Veterinarians.

[28] National Health and Medical Research Committee (2013). Australian Code for the Care and Use of Animals for Scientific Purposes.

[29] British Horseracing Authority BHA Briefing: New Figures Show Whip Offences Continued to Fall in 2015. http://www.britishhorseracing.com/wp-content/uploads/2016/01/BHA-BRIEFING-2015-Whip-Data-14-01-16.pdf.

[30] Murrihy R. (2016). Chief Steward NSW Racing, Sydney, NSW, Australia.

[31] Van Gestel M. (2016). Deputy Chairman of Stewards—Operations, NSW Racing, Sydney, NSW, Australia.

[32] Racing NSW Annual Report 2014. http://www.racingnsw.com.au/site/_content/document/00001257-source.pdf.

[33] Van Gestel M. (2016). Deputy Chairman of Stewards—Operations, NSW Racing, Sydney, NSW, Australia.

[34] Australasian Legal Information Institute Prevention of Cruelty to Animals Act 1979. http://www.austlii.edu.au/au/legis/nsw/consol_act/poctaa1979360/s4.html.

[35] British Horseracing Authority Guide to Procedures and Penalties 2016. http://www.britishhorseracing.com/wp-content/uploads/2014/03/guide-procpen-2016.pdf.

[36] International Federation of Horseracing Authorities International Agreement on Breeding, Racing and Wagering 25 January 2016. http://www.horseracingintfed.com/resources/2015Agreement.pdf.

[37] International Group of Specialist Racing Veterinarians (IGSRV) WhipChip the Electronic Whip. http://igsrv.org/whipchip:-the-electronic-whip.

[38] Law Council of Australia Policy Statement–Rule of Law Principles. http://www.lawcouncil.asn.au/lawcouncil/images/LCA-PDF/a-z-docs/PolicyStatementRuleofLaw.pdf.

[39] McGreevy P.D., Ralston L. (2012). The distribution of whipping of Australian Thoroughbred racehorses in the penultimate 200 m of races is influenced by jockeys’ experience. J. Vet. Behav..

[40] McGreevy P.D., Caspar G.L., Evans D.L. (2013). A pilot investigation into the opinions and beliefs of Australian, British, and Irish jockeys. J. Vet. Behav..

[41] Van Gestel M. (2016). Deputy Chairman of Stewards—Operations, NSW Racing, Sydney, NSW, Australia.

[42] Racing Australia Media Release Letter from Racing Australia Chairman—Sent To All Jockeys Today 19 November 2015. http://www.racingnsw.com.au/default.aspx?s=article-display&id=19005.

[43] Racing Australia Media Release (2015). Whips. http://www.racingaustralia.horse/uploadimg/media-releases/Racing%20Australia%20Media%2023%20October%202015.pdf.

[44] Racenet.com.au (2016). Berry’s Penalty the Severest of Whip Infringers. https://www.racenet.com.au/news/121649/Berry%E2%80%99s-penalty-the-severest-of-whip-infringers.

[45] G1X Stewards to Get Cracking on the Whip Rule. https://www.g1x.com.au/news/racing/stewards-to-get-cracking-on-the-whip-rule.

[46] Racing Australia Media Release 24 June 2016. http://www.racingaustralia.horse/uploadimg/media-releases/Racing%20Australia%20Media%2024%20June%202016.pdf.

[47] Knight P.K., Hamilton N.A. (2014). Handedness of whip use by Australian jockeys. Aust. Vet. J..

[48] Grandin T. (2010). Improving Animal Welfare: A Practical Approach.

