# Supplementary material for: Whip Rule Breaches in a Major Australian Racing Jurisdiction: Welfare and Regulatory Implications
Source: Animals (Basel). 2017 Jan 16;7(1):4. doi: 10.3390/ani7010004 (PMC5295154; doi:10.3390/ani7010004)
Supplement: Supplementary file 1 [file animals-07-00004-s001.pdf]

Supplementary Materials: Whip Rule Breaches in a Major Australian Racing Jurisdiction: Welfare and Regulatory Implications

Jennifer Hood, Carolyn McDonald, Bethany Wilson, Phil McManus and Paul McGreevy

Table S1. Details of tracks, race meetings, races, races with breaches, starts, and starts with breaches for Country, Metropolitan, and Provincial locations.

Race Variables	Country *n* (%)	Metropolitan *n* (%)	Provincial *n* (%)	Total
Tracks	112 (91.80%)	5 (4.10%)	5 (4.10%)	122
Race meetings	540 (68.79%)	111 (14.14%)	134 (17.07%)	785
Races	3731 (66.58%)	839 (14.97%)	1034 (18.45%)	5604
Races with breaches	194 (58.43%)	73 (21.99%)	65 (19.58%)	332
Races with 2 horses with breaches	6	4	4	14
Races with 3 horses with breaches	0	0	1	15
Races with second breaches on same horse	16 (43.24%)	5 (13.51%)	16 (43.24%)	37
Starts	38,020 (67.34%)	8204 (14.53%)	10,232 (18.12%)	56,456
Starts with breaches	200 (57.47%)	77 (22.13%)	71 (20.40%)	348
Starts with second breaches on same horse	16 (43.24%)	5 (13.51%)	16 (43.24%)	37

Table S2. Whip rules breached in second breaches classified by Country, Metropolitan, and Provincial location.

Breach Code	Country Track Number out of 200 (%)		Metropolitan Track Number out of 77 (%)		Provincial Track Number out of 71 (%)	
No second breach	184	(92.00%)	72	(93.51%)	55	(77.46%)
8	2	(1.00%)	1	(1.30%)	2	(2.82%)
16	13	(6.50%)	1	(1.30%)	7	(9.86%)
17	1	(0.50%)	3	(3.90%)	7	(9.86%)

Code 8—Whip use that raises arm above jockey's shoulder height; Code 16—Forehand whip use * in consecutive strides prior to 100 m mark; Code 17—Forehand whip use * on more than 5 occasions prior to 100 m mark; * Whip rules were amended 1 December 2015 to include backhand whip strikes as well as existing forehand restrictions.

Table S3. Fines resulting from first breaches.

Fine $	Number of Fines	Total $
100	12	1200
200	61	12,200
300	16	4800
400	8	3200
500	4	2000
600	1	600
800	2	1600
Total	104	25,600

Table S4. Outcomes of second breaches classified by whip rule breached.

Second Breach Code	C (*n*)	R (*n*)	$200 (*n*)	S (*n*)	CR (*n*)
8	2	3	0	0	0
16	0	16	0	1	4
17	0	6	1	3	1

C—Caution; R—Reprimand; $200—Fine; S—Suspension; CR—Conviction Recorded; Code 8—Whip use that raises arm above jockey's shoulder height; Code 16—Forehand whip use * in consecutive strides prior to 100 m mark; Code 17—Forehand whip use * on more than 5 occasions prior to 100 m mark; * Whip rules were amended 1 December 2015 to include backhand whip strikes as well as existing forehand restrictions.

Table S5. Total prize money on offer in races with breaches by location.

Location	KW chi-sq	Df	*p* Value
Country	3.1522	4	0.5327
Provincial	6.3111	4	0.1771
Metropolitan	10.3853	4	0.0344

Table S6. Length of races with breaches.

Distance	Number	Distance	Number	Distance	Number
**900**	6	**1250**	4	**1580**	1
**950**	1	**1260**	1	**1600**	50
**1000**	25	**1280**	4	**1603**	2
**1006**	1	**1300**	21	**1624**	1
**1010**	1	**1303**	1	**1700**	1
**1012**	1	**1320**	1	**1750**	1
**1080**	1	**1350**	5	**1800**	8
**1100**	23	**1370**	1	**1812**	3
**1106**	3	**1400**	45	**1850**	1
**1120**	1	**1406**	2	**1900**	11
**1150**	2	**1420**	1	**2000**	17
**1175**	1	**1425**	3	**2012**	2
**1180**	2	**1450**	3	**2030**	1
**1200**	45	**1460**	1	**2100**	8
**1206**	2	**1500**	13	**2200**	4
**1207**	1	**1506**	1	**2300**	1
**1215**	1	**1530**	2	**2400**	7
**1240**	1	**1550**	2		

This corresponds to

Min	1st Quartile	Median	3rd Quartile	Max
900	1200	1400	1600	2400

Table S7. Riders with breaches (including repeat offenders).

Race Variables	*n*	%
Sum of starts with breaches	348	
Number of riders	139	
Number of singles	68	48.92
Number of repeats	71	51.08
Number of males	108	77.70
Number of repeat males	62	87.32
Number of females	31	22.30
Number of repeat females	9	12.68
Number of jockeys	91	65.47
Number of repeat jockeys	49	69.01
Number of apprentices	48	34.53
Number of repeat apprentices	22	30.99

Table S8. Riders with the highest numbers of breaches.

Rider Code	Jockey/Apprentice	Gender	Number of Starts with One or More Breaches	Number of Starts with Second Breaches
A	Jockey	Male	13	2
B	Jockey	Male	12	0
C	Jockey	Male	12	2
D	Apprentice	Male	10	2
E	Jockey	Male	9	1
F	Jockey	Male	9	1
G	Jockey	Male	8	3
H	Jockey	Male	8	1
I	Jockey	Male	7	1
J	Jockey	Male	6	1
K	Jockey	Male	6	1
L	Jockey	Male	6	0
M	Jockey	Female	6	0
N	Jockey	Male	6	0
O	Jockey	Male	6	1

Rider code—each letter represents the name of a different rider.

Table S9. Riders breaching whip rules in two races at the same race meeting.

Date 2013	Race Track	Location	Rider Code	Jockey/Apprentice	Gender
9 January	Warwick Farm	M	B	Jockey	Male
6 April	Rosehill	M	G	Jockey	Male
13 April	Port Macquarie	C	H	Jockey	Male
19 April	Ballina	C	P	Apprentice	Male
16 July	Wagga Wagga	C	Q	Apprentice	Male
21 July	Hawkesbury	P	A	Jockey	Male
31 July	Warwick Farm	M	F	Jockey	Male
26 August	Bathurst	C	I	Jockey	Male
31 August	Hawkesbury	P	R	Apprentice	Female
19 September	Newcastle	P	A	Jockey	Male
12 October	Kembla Grange	P	S	Jockey	Male
5 November	Orange	C	O	Jockey	Male
6 December	Coffs Harbour	C	T	Apprentice	Female

M—Metropolitan, C—Country, P—Provincial; Rider code—each letter represents the name of a different rider with A–0 the same as designated in Supplementary Materials Table S8.

S3.9.2. Frequency of Whip Rule Breaches in BOBS Races

Excluding the nine starts with breaches in the ACT, there were 339 starts with at least one breach recorded; 68 (20.06%) of these were BOBS horses in BOBS races; 248 (73.16%) were Non-BOBS horses in BOBS races; and 23 (6.78%) were horses in Non-BOBS races. That is, 93.22% of starts resulting in breaches occurred in BOBS races, compared with the 96.07% of races that were BOBS races.

At C locations, 187 (97.91%) of the 191 starts resulting in a breach or breaches were in BOBS races, which comprised over 99% of races. At M tracks, 62 (80.52%) of 77 starts resulting in a breach or breaches occurred in BOBS races, which comprised 82% of races. At P tracks, 67 (94.37%) of 71 starts resulting in a breach or breaches occurred in BOBS races, which comprised 97.39% of races. These data do not suggest BOBS races are more likely to result in breaches. Further details are provided in Supplementary Materials Table S10.

While 99% of C races were BOBS races, only 17.96% of horses starting in C races were BOBS horses. P races had the highest proportion of BOBS horses starting (34.48%), with 97.38% of races at P tracks being BOBS races. M races were the least likely to be BOBS races, at 82%, and yet 27.85% of M starts were by BOBS horses. This suggests the average number of BOBS horses in a BOBS race varies by location. For further details see Supplementary Materials Table S11.

Table S10. BOBS and Non-BOBS races classified by Country, Metropolitan, or Provincial location.

Location	Non-BOBS Races (% out of 212)		BOBS Races (% out of 5187)		Total (% out of 5399 NSW Races)	
Country	34	(16.04%)	3492	(67.32%)	3526	(65.31%)
Metropolitan	151	(71.23%)	688	(13.26%)	839	(15.54%)
Provincial	27	(12.74%)	1007	(19.41%)	1034	(19.15%)

BOBS—Racing NSW Breeder Owner Bonus Scheme.

Table S11. Percentage of BOBS horses in starts classified by Country, Metropolitan or Provincial location.

Horses	Country Starts	Metropolitan Starts	Provincial Starts
BOBS horses (n)	6455	2285	3528
(%)	(17.96%)	(27.85%)	(34.48%)
All HORSES	35,944	8204	10,232

BOBS—Racing NSW Breeder Owner Bonus Scheme.

Table S12. BOBS status of 339 horses with breaches classified by Country, Metropolitan or Provincial location.

Races	Horses	Country (% out of 191 Starts with Breaches)		Metropolitan (% out of 71 Starts with Breaches)		Provincial (% out of 77 Starts with Breaches)		Total (% out of 339 Starts with Breaches)	
**BOBS races**	BOBS horse	26	−13.61%	20	−25.97%	22	−30.99%	**68**	**−20.06%**
	Non-BOBS horse	161	−84.29%	42	−54.55%	45	−63.38%	**248**	**−73.16%**
**Non-BOBS races**		4	−2.09%	15	−19.48%	4	−5.63%	**23**	**−6.78%**

BOBS—Racing NSW Breeder Owner Bonus Scheme.

S3.9.4. Whip Rule First Breaches in BOBS and Non-BOBS Races

(i) Code 16 First Breaches (Forehand Whip Use in Consecutive Strides prior to 100 m Mark) in BOBS and Non-BOBS Races

A χ^2 value of 14.6925 with an associated p value < 0.01 suggests a breach of Code 16 occurs more frequently among first breaches in Non-BOBS races than in BOBS races.

(ii) Code 17 First Breaches (Forehand Whip Use on More Than Five Occasions prior to 100 m Mark) in BOBS and Non-BOBS Races

A χ^2 value of 2.648 with an associated p value = 0.1037 suggests a breach of Code 17 occurs no more frequently among first breaches in Non-BOBS races than in BOBS races.

Table S13. Whip rules breached in first breaches in BOBS and Non-BOBS races.

Breach Code	BOBS Race				Non-BOBS Race	
	BOBS Horses		Non-BOBS Horses		Number (%) out of 23 Breaches	
	Number (%) out of 68 Breaches		Number (%) out of 248 Breaches			
6	3	−4.41%	7	−2.82%	0	0.00%
7	2	−2.94%	4	−1.61%	0	0.00%
8	20	−29.41%	57	−22.98%	9	−39.13%
9	9	−13.24%	33	−13.31%	0	0.00%
13	0	0.00%	1	−0.40%	0	0.00%
14	0	0.00%	1	−0.40%	0	0.00%
16	3	−4.41%	22	−8.87%	8	−34.78%
17	31	−45.59%	114	−45.97%	6	−26.09%
24	0	0.00%	9	−3.63%	0	0.00%

BOBS—Racing NSW Breeder Owner Bonus Scheme; Code 6—Excessive/unnecessary/improper whip use; Code 7—Whip use forward of horse's shoulder/vicinity of head; Code 8—Whip use that raises arm above jockey's shoulder height; Code 9—Whip use when horse is out of contention; Code 13—Whip use when horse is clearly winning; Code 14—Whip use when horse has no reasonable prospect of improving/losing position; Code 16—Forehand whip use * in consecutive strides prior to 100 m mark; Code 17—Forehand whip use * on more than 5 occasions prior to 100 m mark; Code 24—Whip Rule breach not specified by Stewards; * Whip rules were amended 1 December 2015 to include backhand whip strikes as well as existing forehand restrictions.

© 2017 by the authors; licensee MDPI, Basel, Switzerland. This article is an open access article distributed under the terms and conditions of the Creative Commons by Attribution (CC-BY) license (http://creativecommons.org/licenses/by/4.0/).
